# Transitions for older people with learning disabilities and behaviours that challenge others, and their family carers: a merged protocol for two rapid scoping reviews of evidence

**DOI:** 10.1186/s13643-021-01883-3

**Published:** 2022-01-18

**Authors:** J. Vseteckova, J. Jordan, E. Tilley, M. Larkin, S. Ryan, L. M. Wallace

**Affiliations:** 1 School of Health, Wellbeing and Social Care, Faculty of Wellbeing, Education and Language Studies, The Open University, Walton Hall, Milton Keynes, MK7 6AA UK; 2 Department of Social Care and Social Work, Manchester Metropolitan University, Manchester, UK

**Keywords:** Older people, Learning disabilities, Behaviour that challenges others, Family carers, Transition, Forward planning, Scoping review, Rapid review

## Abstract

**Background:**

There are over 1 million adults with a learning disability in the UK, of whom approximately 20% displaying behaviours that challenge others. Two thirds of people with learning disabilities live in the family home. As they and their family carers age, both are likely to face particular difficulties and stresses, but there is little understanding of their experiences and needs. To address this evidence gap, our main objective is to undertake two rapid scoping reviews that will collectively focus on the health and social care needs, experiences, service interventions and resources of older people with learning disabilities and behaviours that challenge others, and their family carers. Both reviews will focus on issues relating to forward planning and transitions to different care contexts. The study is part of a research project funded by the National Institute for Health Research No.129491.

**Methods:**

We propose to address the need for evidence via two rapid scoping reviews. We will include published and unpublished (grey) literature, encompassing empirical research, policy and practice guidance and lay resources to support decision-making. We will search multiple electronic databases, hand search references lists, and use expert guidance to identify potential evidence. The following databases were used for research and grey literature: CINAHL; Healthcare Management Information Consortium (HMIC); NHS Evidence; Scopus; Turning Evidence Into Practice (TRIP); Web of Science (WoS); Google (first 5 pages); and Google Scholar (first 5 pages). For RR2, additional intended databases are the Carer Research Knowledge Exchange Network (CAREN) and Social Care Institute for Excellence (SCIE). Two reviewers will independently screen all citations and full-text articles for inclusion. One reviewer will extract data, with an independent review undertaken by the research team. Critical appraisal will depend on the nature of included evidence. Narrative synthesis will be collaboratively developed, with descriptive information presented in tables summarising study characteristics and thematic analysis of findings presented in the main text. Dissemination will be through journal publication, conference presentations and written short-form, easy-read versions of articles and audio-video clips for lay audiences.

**Discussion:**

We will consider the strengths and limitations of our reviews, considering their impact on findings. We will summarise the main findings and provide an interpretation linked to the review questions and objectives. We will consider the implications of our findings for policy and practice, as well as future research addressing the support of older people with learning difficulties and behaviours that challenge others, and their family carers, in the context of transition to different care contexts in the UK.

The protocol has been registered as Vseteckova, J., Jordan, J., Tilley, E., Larkin, M., Ryan, S., and Wallace, L. (2021, December 4). Transitions for older people with learning disabilities and behaviours that challenge others, and their family carers: a merged protocol for two rapid scoping reviews of evidence. Retrieved from osf.io/jzrn9.

**Supplementary Information:**

The online version contains supplementary material available at 10.1186/s13643-021-01883-3.

## Background

There are approximately 1,130,000 adults with a learning disability in the UK [1], of whom 20% are estimated to engage in behaviours that challenge others [2]. Two thirds of adults with learning disabilities live in the family home, usually with their parents [3]. The life expectancy of people with a learning disability has been increasing, with the population of older people with learning disabilities set to increase four times faster than the overall adult learning disability population [4]. It is estimated that the number of people with learning disabilities using adult social services in the UK will double by 2030 [5]. There is also a growing ‘hidden’ population of people with learning disabilities, many of whom do not become known to services until later in their lives, having been cared for by family members until they become too frail to do so [6–8]. These demographic changes are expected to create substantial pressure on already underfunded services [9], which has not yet been fully quantified [3].

The consequences of ageing for family carers and the older people with learning disabilities for whom they care are deeply entwined. Both face the challenges typically associated with ageing. In addition, people with learning disabilities are more likely to experience early onset of chronic health conditions such as kidney disease, dementia, constipation and diabetes than their non-disabled peers [10, 11], which can remain unaddressed if families and practitioners fail to recognise the symptoms [9]. In some families, mutual caring relationships and dependency develop, where the person with learning disabilities assumes caring responsibilities for an older frail parent [9]. As parental frailty increases, older people with learning disabilities can be left at risk of a breakdown in support [2]. The death of a family member, particularly the main caregiver, can trigger complicated grieving, behaviours that challenge others and the need for crisis intervention [12], in part because parental loss is often accompanied by further losses, including the loss of home [13].

The issue of breakdown in support is compounded by a reluctance on the part of family carers to plan for transition, leaving older people with learning disabilities at risk of inappropriate relocation to more intensive supported care [8, 14, 15]. The majority of family carers wish their adult family member to reside in the family home [16–18], as do a majority of adults with a learning disability PWLD [19–21]. Family carers find making plans for the possible/future transition of their family member to other accommodation extremely challenging. This is in part because they lack confidence in the available alternatives [22–24] and find it difficult to engage with the emotive and complex consideration of their family member’s departure from the home [6, 23, 25]. Transition planning is also made more difficult by a care system that is complex to navigate, and a lack of professional input to guide and support family carers through the process [26–28].

Despite the clear challenges and stresses faced by older people with learning disabilities and behaviours that challenge others and their family carers, we know little about their transition-related experiences and needs. Two systematic reviews addressed people with learning disabilities and behaviours that challenge others, but they focused on younger adults [29, 30]. In addition, the systematic review supporting the National Institute for Clinical Excellence (NICE) guidance on care and support of people growing older with learning disabilities (NG96) [3] does not include a focus on behaviours that challenge others. In relation to ageing carers of older people with learning disabilities and behaviours that challenge others, again very little evidence exists. Systematic reviews that have included such a focus have not dealt specifically with the experiences and needs of older carers of adults with learning difficulties and behaviours that challenge others [26–28].

To address the above gaps in evidence, we will undertake two rapid scoping reviews. The first (RR1) will address older people with learning disabilities and behaviours that challenge others, with a specific focus on ageing, including issues relating to transition. The second (RR2) will address their family carers, again with a specific focus on ageing and issues relating to transition. Both reviews will encompass literature concerning the health and social care needs, experiences, service interventions and resources of and for both populations.

For RR1 our review question is: *What are the health and social care needs, experiences, service interventions and resources of and for older people with learning disabilities and behaviours that challenge others as they move to different care contexts*^1^*in the UK?* In line with this question, our objectives are to (1) Identify relevant UK evidence according to key features such as nature, focus, content, target population, design, methodology and findings/outcomes; (2) Systematically integrate this evidence in terms of what it suggests are the health and social care needs, experiences, service interventions and resources of and for older people with learning disabilities and behaviours that challenge others as they transition to different contexts of care; and (3) Use the learning delivered by (2) to consider the status of transition-related care and support for older people with intellectual disabilities and behaviours that challenge others, drawing out implications for how this care and support might be most effectively planned and undertaken to fit with people’s needs and preferences.

For RR2 our review question is: *What are the health and social care needs, experiences, service interventions and resources of and for family carers*^2^*of older people with learning disabilities and behaviours that challenge others as they move to different care contexts in the UK?* In line with this question, our objectives are to (1) Identify relevant UK evidence according to key features such as nature, focus, content, target population, design, methodology and findings/outcomes; (2) Systematically integrate this evidence in terms of what it suggests to be the health and social care needs, experiences, service interventions and resources of and for family carers of older people with learning disabilities and behaviours that challenge others as they transition to different contexts of care; and (3) Use the learning delivered by (2) to consider the status of transition-related care and support for family carers of older people with intellectual disabilities and behaviours that challenge others, drawing out implications for how this care and support might be most effectively planned and undertaken to fit with people’s needs and preferences.

## Review design/methods

The rationale for undertaking rapid scoping reviews of the literature is as follows. Our review questions are inclusive and exploratory in nature, designed to capture a broad range of evidence pertaining to multiple aspects of the health and social care needs, experiences, service interventions and resources of and for older people with learning disabilities and behaviours that challenge others as well as exploring multiple aspects of the health and social care needs, experiences, service interventions and resources of and for the family carers. Scoping reviews seem ideally suited to our requirements, as it allows evidence drawn from diverse sources, which is typically heterogeneous in nature, to be systematically synthesised in terms of its nature, features, and findings/outcomes [31]. Given that these rapid reviews constitute the first stage of a much broader study^3^, it is important that it is completed in a timely manner. In such circumstances, a rapid review is recommended [32]. Rapid reviews are a streamlined and/or accelerated version of fully systematic reviews [33] and have become an increasingly accepted approach to the generation of evidence [34, 35]. Estimates vary on the length of time required for completion [33], but they are typically understood to take 6 months or less [33].

This study protocol has been registered in the Open Science Framework (osf.io/jzrn9); it is publicly available for view in the National Institute of Health Research (NIHR) library. It is being reported in accordance with the reporting guidance provided in the Preferred Reporting Items for Systematic Reviews and Meta-Analyses Protocols (PRISMA-P) statement [36] (see checklist in Additional file 1). The planned reviews will be reported according to the Preferred Reporting Items for Systematic reviews and Meta-Analyses extension for Scoping Reviews (PRISMA-ScR) Checklist [37]. We will also draw on relevant expert guidance. We will use the *S*elec*T*ing *A*pproaches for *R*apid *R*eviews (STARR) [38] decision tool to help make broad decisions concerning the overall review process. In terms of specific methods/techniques, we will draw on guidance from the Oxford Centre for Evidence Based Medicine [35] and the World Health Organization (WHO) [32].

The guidance for rapid reviews repeatedly stresses the importance of interaction with the users of the review to ensure it remains relevant and useful [34, 35]. We will consult with our expert advisory groups, which include people with learning disabilities, family carers, health and social care professionals, policy-makers, commissioners and service providers. The groups will guide the review at all stages, to contribute ideas, discuss ongoing findings, and help ensure clarity and relevance of analysis. This necessary extensive user/expert involvement carries significant resource implications [35]. In order to ensure adequate time to complete such preparatory steps, as well as undertake a robust and properly considered review, we anticipate that both reviews will be completed within 6 months.

### Eligibility criteria

Given that we aim to scope a broad range of evidence in RR1 and RR2, we will include published and unpublished (grey) literature, including research articles, reports and guidance. Whilst we will include policy and practice guidance, we will exclude discussion papers, position papers, expert opinion pieces, editorials and study protocols as we are interested in the nature of and findings of evidence that can be used to draw conclusions regarding our phenomena of interest.

Within the published research we will include both primary (using quantitative, qualitative and mixed methods) and secondary (e.g. review) level evidence. Given our time and other resource constraints, we will only include literature written in English. To enhance the relevance of our review findings, we will include evidence made available after 2001, to coincide with the publication of the UK Government’s *Valuing People* White Paper for England and Wales [39]. *Valuing People* included an explicit focus on the needs of older people with learning disabilities and on the needs of people with behaviours that challenge others. For both reviews, where evidence encompasses countries in addition to the UK, it will be included if the UK specific evidence can be extracted e.g. when we can differentiate between data in primary research findings or we can use UK-only references included in systematic reviews.

Table 1 sets out the focus of RR1 and RR2, using the Population, Concepts and Context (PCC) framework [40].

**Table 1:** Focus of RR1 and RR2

	Population	Concepts	Context
**RR1**	Older (40+)^a^ adults with learning disabilities and behaviours that challenge others.	Health and social care needs, experiences, service interventions and resources^b^ of and for these older adults.	Older (40+) adults with learning disabilities and behaviours that challenge others transitioning to different contexts of care.
**RR2**	Family carers of older (40+) adults with learning disabilities and behaviours that challenge others.	Health and social care needs, experiences, service interventions and resources of and for these family carers.	Family carers of older (40+) adults with learning disabilities and behaviours that challenge others transitioning to different contexts of care.

^a^Our rationale for defining ‘older adults with learning disabilities and behaviours that challenge others’ as 40+ in this context is twofold. 40+ for people with learning disabilities has been selected to reflect the early onset of some chronic health conditions, such as dementia, for this group.^10^ In addition, defining 40+ enables us to include more family carers who might be described as ‘older’ and in need of new/additional support—i.e. family carers in their early 60s+

^b^Our definition of ‘resources’ encompasses (written) guidance and practical tools publicly available in hard copy format and/or online

Table 2 sets out other inclusion criteria to be used for RR1 and RR2.

**Table 2:** Other inclusion criteria for RR1 and RR2

	Other inclusion criteria
**RR1**	• Be published in English • Be published/made available after 2001 • Concern older (40+) adults with learning disabilities and behaviours that challenge others resident in the UK • Concern these adults in the context of their move between different contexts of care e.g.: ◦ From family care to service care; ◦ From one form of service care to another e.g. supported living to residential/nursing home care; residential care to nursing home care; ◦ From one version of family care (e.g. parent-led) to another (e.g. sibling-led) • Report empirical research focused on health and social care needs and experiences • Report a systematic review of empirical research focused on health and social care needs and experiences • Report service interventions targeting health and social care needs • Report resources relevant to health and social care needs.
**RR2**	• Be published in English • Be published/made available after 2001 • Concern unpaid family carers (e.g. parents, siblings) resident in the UK who provide care to adults (aged 40+) with learning disabilities and behaviours that challenge others • Concern these family carers in the context of their adult family member’s move between different contexts of care e.g.: ◦ From family care to service care; ◦ From one form of service care to another e.g. supported living to residential / nursing home care; residential care to nursing home care; ◦ From one version of family care (e.g. parent-led) to another (e.g. sibling-led) • Report empirical research focused on health and social care needs and experiences • Report systematic reviews of empirical research focused on health and social care needs and experiences • Report service interventions targeting health and social care needs • Report resources relevant to health and social care needs.

### Outcomes and prioritisation

For RR1, we expect the following outcomes:

- Primary: to identify empirical research which indicates the enablers and barriers (systems, processes, commissioning arrangements, workforce skills, and individual/family experience) that either facilitate or hinder effective planning for, and execution of, transition of older adults with learning disabilities and behaviours that challenge others in relation to their personal needs;
- Secondary: to identify resources/tools/models/approaches that provide transition-related information and support to this population.

For RR2, we expect the following outcomes:

- Primary: to identify empirical research which indicates the enablers and barriers (systems, processes, commissioning arrangements, workforce skills, carers’ personal challenges and individual/family engagement) that either support or hinder effective transition planning by carers for this population as they age;
- Secondary: to identify resources/tools/models/approaches in the literature that engage with, and provide information and support to, this population (and those who care for them) as they age.

### Information sources and search strategy

All database selection, search strategies and searches will be undertaken with the support of a subject specialist librarian. Given the short timescale and consequent need to achieve a balance between sensitivity and specificity^4^, we will focus on priority information sources. For both reviews, intended databases (from inception onwards) covering both published and grey literature are: CINAHL; Healthcare Management Information Consortium (HMIC); NHS Evidence; Scopus; Turning Evidence Into Practice (TRIP); Web of Science (WoS); Google (first 5 pages); and, Google Scholar (first 5 pages). For RR2, additional intended databases are: the Carer Research Knowledge Exchange Network (CAREN) and Social Care Institute for Excellence (SCIE) databases may be of value. Final decisions on databases to be included will be made using the “Healthcare Databases Advanced Search” (part of NHS Evidence) resource as a means of determining those most likely to yield relevant evidence. We will use the expertise of the project research team and project advisory group and hand search the reference lists of all included documents to identify any additional evidence sources.

For electronic databases, we will generate search terms (words and phrases, including synonyms and terminology variations). These terms will be combined using the Boolean operators ‘and/or’ and appropriate truncation and phrase symbols to form initial search strategies, which we will pilot against selected key databases. On the basis of this exercise, we will confirm our final search strategies to be used for each of the databases. A draft search strategy for SCOPUS is provided in Additional file 2. We will use search terms similar to our draft Scopus search to find articles for inclusion. The same keywords for the main search will be used to search grey literature each time.

### Selection of sources of evidence

Electronic search datasets will be imported into Excel and duplicate records removed prior to screening. For RR1 and, separately, for RR2, two reviewers will independently screen all returned titles and abstracts (where available) against the inclusion criteria. Through this process we will:

- Exclude articles/reports etc. that clearly do not meet the inclusion criteria
- Identify articles/reports etc. for full paper review.

Any discrepancies will be discussed between the reviewers in the first instance and, if necessary, with the wider review team. The emphasis will be on inclusivity at this stage, so that we will retain those articles/reports etc. about which no firm decision can be made on the basis of the titles/abstracts.

Full-text copies of potentially relevant articles/reports etc. will be obtained. The record containing the most complete data on any single study/other source of evidence will be identified as the primary source of evidence (usually the original study or most recent report). Two reviewers will independently review all full texts for RR1, two reviewers will independently review all full texts for RR2. In order to reduce the potential for bias, and to promote transparency and consistency, a standardised tool will be used (see Additional file 3). Any discrepancies will be discussed between the reviewers in the first instance and, if necessary, with the wider review team. All articles / other sources of evidence excluded on the basis of full-text review will be recorded, alongside the reasons for exclusion, in a Table of Excluded Studies. In cases where evidence is not immediately available, we will attempt to source it using various means (e.g. contacting relevant authors). Given time constraints, if the evidence does not become available within a 1-month period, it will be recorded as “missing”.

### Data extraction

Dedicated data extraction forms will be developed for RR1 and RR2, based on the objectives of each review and consequent categories of information/evidence of interest. They will be piloted independently by one reviewer on three included sources of evidence, selected to ensure variation in focus and content. The form will be revised, as necessary, to ensure it reliably interprets and captures all relevant data from all study designs, report and guideline formats.

The following categories of information are likely to be extracted: authors; year of publication; type of evidence (e.g. research article, practice guidelines); funding/sponsoring organisation; geographical area of the UK; aims and objectives; study/service intervention design; nature of resource; study participants/service intervention population; sample size; study research methods; and, results/findings/outcomes/other content. Additional/amended categories will be confirmed in discussion with the expert advisory group, once the nature of the available evidence is known.

Using the final form, one reviewer will independently extract data from included articles / other sources of evidence, separately for RR1 and RR2. The same reviewer (for consistency purposes) will then collate the information from all of the forms onto a series of Excel master sheets. Each sheet will include the information extracted for each category for all included articles/other sources of evidence. Any uncertainty regarding the data to be extracted will be resolved through discussion within the review team. Where data is missing, we will attempt to contact relevant authors. Given our time constraints, if an author is uncontactable after two contact attempts over a 1-month period, we will record the data as “missing”. All completed data extraction forms will be independently reviewed against relevant texts by review team members as a means of checking for gaps and errors.

### Critical appraisal of individual sources of evidence

The conduct of critical (quality) appraisal in both scoping and rapid reviews is generally considered optional [32, 33, 40]. For scoping reviews, the central issue concerns the inclusion of many types of evidence [40] and for rapid reviews, it concerns both the nature of the evidence and the time available [34]. Final decisions concerning if and how the appraisal is undertaken and how the findings of this appraisal inform the exclusion of evidence will be made once we have a clearer idea of the volume and nature of the included evidence. If the quality appraisal is undertaken, it is likely to employ relatively straightforward and/or higher-order approaches, as per the recommendations [35]. It will include the same processes of checking for missing information and of independent verification as outlined for other stages of our review.

### Synthesis of findings

Given the broad scope of our research question, the included evidence will be diverse in nature. In both reviews, alongside empirical research findings, our evidence is likely to include policy and practise guidelines relevant to our populations, as well as lay resources to support transition-related decision-making. Such diversity will necessitate a flexible, yet robust, approach to bringing together the body of evidence in its entirety. Although decisions concerning our detailed approach to synthesis can be made only when the precise nature of available evidence is known, it is possible to provide an outline at this stage.

For each review, we will include a PRISMA flow diagram [41], outlining the: number of sources of evidence screened; the number of sources of evidence subject to full-text review, with reasons for exclusion; and, number of sources included in the review. For each review, we will summarise the key characteristics of included evidence in a Table of Characteristics. Using the evidence included in the table, we will identify patterns and trends in the volume, focus and content of included evidence, as a basis of narrative comment in the Discussion sections of the reviews. We will integrate the findings/content of the included evidence in narrative form, drawing on expert guidance on the conduct of narrative synthesis in systematic reviews [42, 43], commonly used when evidence is derived from studies using a range of research designs and methods. This approach will enable the identification of overarching themes according to the essential meaning of the collective bodies of evidence, however derived and expressed.

Analysis will be led by one reviewer for consistency. To promote rigour, early drafts will be shared with the review team, who will provide feedback on fit with the original data, as well as overall sense and insight provided. Successive drafts of the synthesis will be shared with our project advisory group for ongoing discussion, review and refinement. This process will enable the production of analyses, which have benefited from the input of a range of expert knowledge and understandings.

## Discussion

We have assembled a research team to collectively undertake the reviews, with designated roles and responsibilities, and so do not anticipate any adverse operational issues in respect of their completion. Practically, we may encounter difficulties in accessing potential sources of evidence in the time frame within which we are operating (a rapid review), but have sought to mitigate these by imposing an explicit approach (e.g. imposing a clear timeline, and criteria for designating potential sources of evidence as ‘missing’). As a rapid scoping review, the possibility that we will miss relevant sources of evidence remains. We will make strenuous efforts to capture all relevant sources, not only through our database searching and hand searching of reference lists, but also through the advice and direction from our Project Advisory Group, which is wide-ranging in representation (see below). From our initial scan of the literature, relevant sources of evidence appear limited; it is therefore all the more vital to identify what does exist as a means of identifying gaps as this will enable informed decision-making about future research and the development of practical resources to aid the effective transition.

For each RR1 and RR2, we will summarise the main findings (including an overview of concepts, themes, and types of evidence available). We will consider the strengths and limitations of the included evidence, as these impact on the findings of the review. We will also consider the strengths and limitations of our review methodology, again considering their impact on review findings. We will provide an overall interpretation of review findings with respect to their respective questions and objectives. We will also consider the implications of our findings for policy and practice, as well as future research.

All aspects of the reviews will be set out in detailed main publications. This will ensure that the reviews are transparent, reproducible, and could be updated in the future [34]. Due to the iterative nature of the reviews, there may be deviations from the protocol; these will be described and justified in the full paper publications. At this stage, we do not have plans to update RR1 and RR2 once completed, but will consider this possibility as our overall study unfolds.

Expert guidance stresses a need for rapid reviews to be reported clearly and communicated in a way that fits the practical needs and context of knowledge users [32, 34]. Both reviews will be of interest to a range of audiences (e.g. people with learning disabilities, family carers, service providers, policy-makers and commissioners). We will consider how best to develop summaries according to their specific requirements. Here, the fact that these audiences are represented on our project advisory group will be of considerable value. Our plans for dissemination include publication of both reviews in academic journals, and additional publications/resources with user-friendly plain-language summaries, Easy Read or infographics and OpenLearn articles. Given the Open University’s expertise in this area—developing innovative, high-quality and accessible materials—we will be able to draw on bespoke support. All outputs will be hosted on diverse, open-access platforms.

## Supplementary Information

**Additional file 1.** PRISMA-P 2015 Checklist.

**Additional file 2.** Draft Scopus Search Strategy.

**Additional file 3.** RR1: Study inclusion screening form. RR2: Study inclusion screening form.

## Data Availability

All search strategies and the data extraction forms are available from the corresponding author, on request.

## Abbreviations

NHS: National Health Service
NICE: National Institute for Clinical Excellence
NIHR: National Institute for Health Research
NIHR, HS&DR: National Institute for Health Research, Health Services & Delivery Research
PRISMA: Preferred Reporting Items for Systematic Reviews and Meta-Analyses
PRISMA-P: Preferred Reporting Items for Systematic Reviews and Meta-Analyses- extension for Protocols
PRISMA-ScR: Preferred Reporting Items for Systematic Reviews and Meta-Analyses- extension for Scoping Reviews

## Footnotes

1 We adopt a wide-ranging definition of ‘care contexts’ for the purpose of this review, which encompasses the following environmental factors: service type (is the person living in a congregate setting, independently, or in a family home); provider type (NHS/local authority, private provider, third sector organisation, family care); relationships (who provides the care e.g. different family members; paid carers; personal assistants); place (the geographical location of care, taking particular note of the urban/rural dimension); and commissioning and funding arrangements. Our definition of care contexts also takes into account the dynamic and fluid ways in which different contextual factors interact at the micro, meso and macro levels [30].

2 We have opted for the term ‘family carers’ in this protocol, as it reflects the terminology used in the current academic, policy and practice literature. We include parents and siblings in our definition. However, we appreciate that this term can be contentious.

3 For details of the study “Improving the support for older people with learning disabilities and behaviours that challenge others, family and professional carers, and end of life care planning for carers” (HS&DR - NIHR129491), please visit: https://www.journalslibrary.nihr.ac.uk/programmes/hsdr/NIHR129491/#/.

4 Sensitivity is necessary for completeness and specificity is needed for manageability.
