# Supplementary material for: Transitions for older people with learning disabilities and behaviours that challenge others, and their family carers: a merged protocol for two rapid scoping reviews of evidence
Source: Syst Rev. 2022 Jan 18;11:14. doi: 10.1186/s13643-021-01883-3 (PMC8767693; doi:10.1186/s13643-021-01883-3)
Supplement: Supplementary file 2 — Additional file 2. Draft Scopus Search Strategy. [file 13643_2021_1883_MOESM2_ESM.docx]

**Draft Scopus Search Strategy**

TITLE-ABS(( ( "challeng* behav*" ) OR ( behav* W/5 challeng* ) OR ( "problem* behav*" ) OR ( aggression ) OR ( aggressive W/5 behav*) OR ( "behav* of concern" ) OR ( risky W/5 behav* ) OR ( "high risk" W/5 behav* ) OR ( "self-injur*" ) OR ( "self-harm*" ) OR ( antisocial W/5 behav* ) OR ( anti-social W/5 behav* ) OR ( unacceptable W/5 behav* ) OR ( inappropriate W/5 behav* ) OR ( worrying W/5 behav* ) ) AND ( ( ( "developmental* disab*" ) OR ( "developmental delay" ) OR ( "learning disab*" ) OR ( "intellect* disab*" ) OR ( "intellect* disorder*" ) OR ( "intellect* impair*" ) OR ( "intellect* handicap*" ) OR ( "learning difficult*" ) OR ( "cognitive disab*" ) OR ( "cognitive impair*" ) OR ( "mental retard*" ) OR ( "mental handicap*" ) ) AND ( ( "older adult*" ) OR ( aging ) OR ( ageing ) OR ( elderly ) OR ( senior* ) OR ( "adult child*" ) OR ( "old age" ) OR ( "middle age*" ) OR ( frail* ) OR ( midlife ) OR ( "mid-life" ) OR ( "middle life" ) OR ( "late* life" ) OR ( "aged adult*" ) OR ( "mature adult*" ) ) ) ) Limit to SCOPUS's UK geographical filter
