# Supplementary material for: Transitions for older people with learning disabilities and behaviours that challenge others, and their family carers: a merged protocol for two rapid scoping reviews of evidence
Source: Syst Rev. 2022 Jan 18;11:14. doi: 10.1186/s13643-021-01883-3 (PMC8767693; doi:10.1186/s13643-021-01883-3)
Supplement: Supplementary file 3 — Additional file 3. RR1: Study inclusion screening form. RR2: Study inclusion screening form. [file 13643_2021_1883_MOESM3_ESM.docx]

**RR1: Study inclusion screening form**

| What are the health and social care needs, experiences, service interventions and resources of and for older people with learning disabilities and behaviours that challenge others as they move to different care contexts in the UK? | |
| --- | --- |
| Study ID (family name of first author & year of publication + letter if more than one per year e.g. Smith 2019a) |  |
| Form completed by |  |
| Date of completion |  |
| Are there any other records of the same study? Yes/No/Unclear |  |
| Criterion | Yes/No/Unclear, with explanation if required |
| 1. Be published in English |  |
| 2. Be published / made available since 2001 |  |
| 3. Concern older (40+) adults with learning disabilities and behaviours that challenge others resident in the UK |  |
| 4. Concern older adults with learning disabilities and behaviours that challenge others who are moving to different care contexts |  |
| ***All of the answers to 1., 2., 3. and 4. must be Yes to Include*** |  |
| 5. Report empirical research focused on health and social care needs and experiences |  |
| 6. Report a systematic review of empirical research focused on health and social care needs and experiences |  |
| 7. Report service interventions targeting health and social care needs |  |
| 8. Report resources relevant to health and social care needs |  |
| ***At least one of the answers to 5., 6., 7. and 8. must be Yes to Include*** |  |
| **Decision - Include / Exclude / More information needed before decision can be made**  **(*please specify information required*)** |  |

**RR2: Study inclusion screening form**

| What are the health and social care needs, experiences, service interventions and resources of and for family carers of older people with learning disabilities and behaviours that challenge others as they move to different care contexts in the UK? | |
| --- | --- |
| Study ID (family name of first author & year of publication + letter if more than one per year e.g. Smith 2019a) |  |
| Form completed by |  |
| Date of completion |  |
| Are there any other records of the same study? Yes/No/Unclear |  |
| Criterion | Yes/No/Unclear, with explanation if required |
| 1. Be published in English |  |
| 2. Be published / made available since 2001 |  |
| 3. Concern unpaid family carers (e.g. parents, siblings) resident in the UK who provide care to adults (aged 40+) with learning disabilities and behaviours that challenge others |  |
| 4. Concern unpaid family carers of adults with learning disabilities and behaviours that challenge others who are moving to different care contexts |  |
| **All of the answers to 1., 2., 3. and 4. must be Yes to Include** |  |
| 5. Report empirical research focused on health and social care needs and experiences |  |
| 6. Report a systematic review of empirical research focused on health and social care needs and experiences |  |
| 7. Report service interventions targeting health and social care needs |  |
| 8. Report resources relevant to health and social care needs. |  |
| **At least one of the answers to 5., 6., 7. and 8. must be Yes to Include** |  |
| **Decision - Include / Exclude / More information needed before decision can be made (please specify information required)** |  |
